# ^192^Ir brachytherapy combined with external beam radiotherapy and biliary stenting for unresectable hilar cholangiocarcinoma

**DOI:** 10.3389/fonc.2026.1909866

**Published:** 2026-08-05

**Authors:** Wenbo Yang, Yinghao Wang, Li Xiao, Fei Liu, Jianxi Zhou, Yunchuan Sun

**Affiliations:** 1 Department of Head, Neck and Thoracic Medical Oncology, Cangzhou Hospital of Integrated Traditional Chinese and Western Medicine-Hebei, Cangzhou, Hebei, China; 2 Department of Second General Surgery, Hengshui Fifth People’s Hospital, Hengshui, Hebei, China

**Keywords:** biliary stenting, brachytherapy, external beam radiotherapy, hilar cholangiocarcinoma, iridium-192

## Abstract

**Objective:**

To evaluate the efficacy and safety of ^192^Ir brachytherapy combined with external beam radiotherapy (EBRT) and biliary stenting in patients with unresectable hilar cholangiocarcinoma.

**Methodology:**

Clinical data of 62 patients with unresectable hilar cholangiocarcinoma were retrospectively analyzed. All patients received concurrent chemoradiotherapy plus percutaneous biliary stenting. Among them, 32 patients (^192^Ir group) additionally received ^192^Ir brachytherapy, and 30 patients (EBRT group) received EBRT alone. Survival outcomes were analyzed using the Kaplan–Meier method.

**Results:**

At 3 months after treatment, the objective response rate was 93.8% in the ^192^Ir group and 70.0% in the EBRT group. Median progression-free survival was 11.2 months vs 8.6 months (P = 0.032), and median overall survival was 14.5 months vs 12.2 months (P = 0.361). Liver function parameters and CA19–9 levels decreased in both groups, with significantly lower levels in the ^192^Ir group at 3 months. Adverse events were tolerable in both groups.

**Conclusion:**

^192^Ir brachytherapy plus EBRT and biliary stenting may improve short-term tumor response and progression-free survival in unresectable hilar cholangiocarcinoma, with tolerable toxicity.

## 1 Introduction

Cholangiocarcinoma is classified into intrahepatic cholangiocarcinoma, hilar cholangiocarcinoma, and distal cholangiocarcinoma. Hilar cholangiocarcinoma arises from the mucosa of the common hepatic duct, left and right hepatic ducts, and their confluence, accounting for 60% to 70% of extrahepatic cholangiocarcinomas. The incidence of hilar cholangiocarcinoma shows geographic variation, with a rising trend in global prevalence in recent decades (1, 2). Surgical resection is the only potentially curative treatment for long-term survival (3). The 5-year survival rate after curative resection of hilar cholangiocarcinoma is approximately 12% (4, 5), while 60% to 70% of patients are diagnosed at locally advanced stages and lose the opportunity for surgery (6).

For patients with unresectable locally advanced hilar cholangiocarcinoma, systemic therapies including chemotherapy, targeted therapy, and immunotherapy, along with locoregional interventions, may provide clinical benefit (7–10). External beam radiotherapy alone is often limited by surrounding organs at risk, which prevents delivery of tumoricidal doses and may lead to local recurrence or disease progression (11). In clinical practice, combined interventional therapy, brachytherapy, and external beam radiotherapy are applied to enhance local control of hilar cholangiocarcinoma. Data on brachytherapy combined with external beam radiotherapy and biliary stenting for this patient population remain limited.

^192^Ir high-dose-rate brachytherapy delivers rapid dose falloff around the source, allowing dose escalation to the tumor target while reducing exposure to adjacent organs at risk. It is commonly used as a boost modality following external beam radiotherapy to enhance local tumor control. Placement of biliary stents can effectively relieve biliary obstruction and reduce jaundice. In this study, we retrospectively analyzed clinical data of patients with unresectable locally advanced hilar cholangiocarcinoma treated with ^192^Ir brachytherapy plus external beam radiotherapy and biliary stenting in our hospital, to evaluate the efficacy and safety of this regimen and explore its clinical application value.

## 2 Materials and methods

### 2.1 Patient information

**Figure 1:**
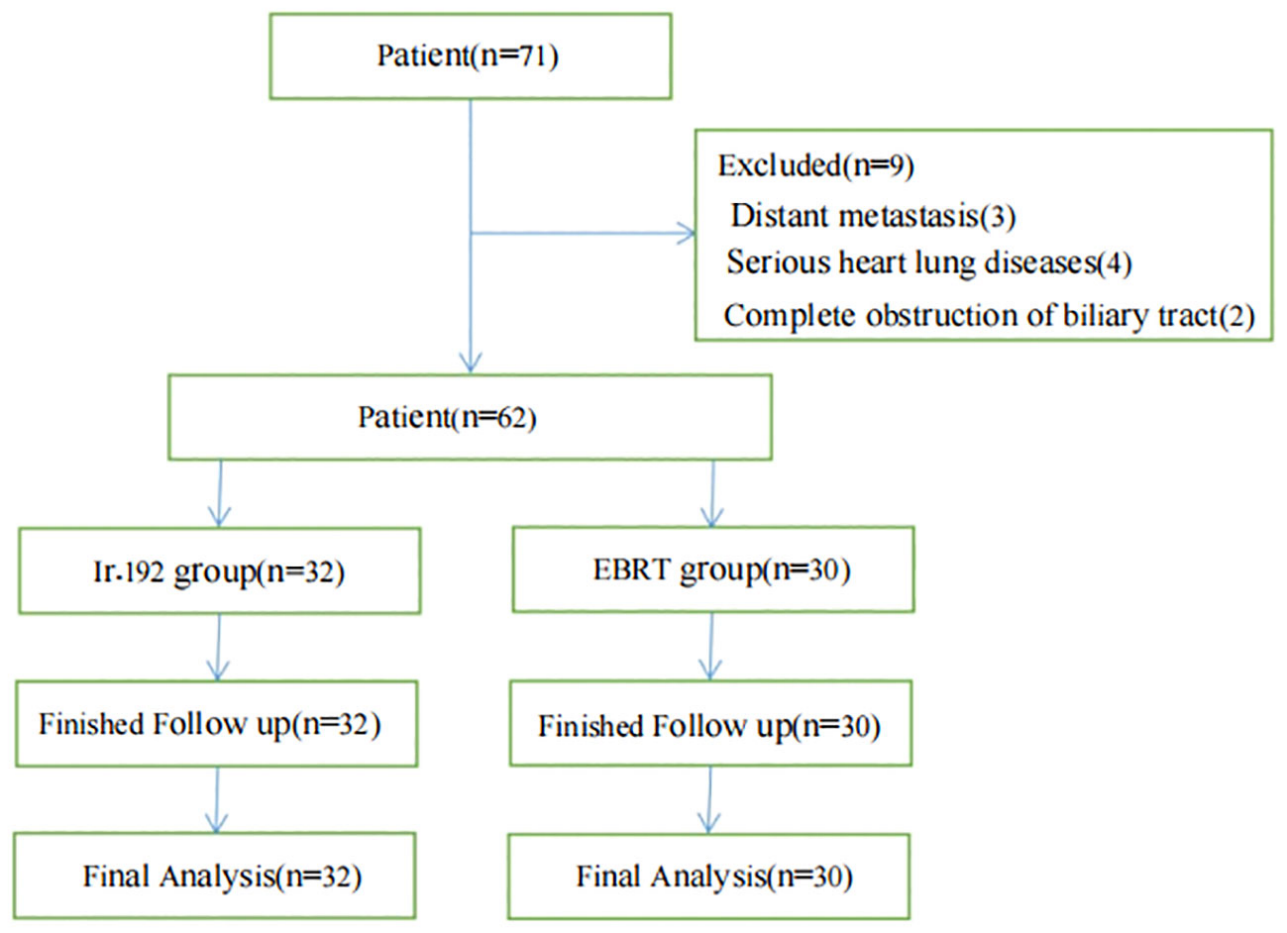
Flowchart of patient enrollment and follow-up.

A total of 71 patients with hilar cholangiocarcinoma indicated for radiotherapy, diagnosed by pathology or imaging, were initially screened at our hospital from January 2014 to March 2019. Three patients with distant metastasis, four patients with severe cardiopulmonary dysfunction, and two patients with complete biliary obstruction where guidewire passage and stent implantation were not feasible were excluded. Finally, 62 patients with unresectable hilar cholangiocarcinoma were included for analysis (**Figure 1**). The study was approved by the institutional ethics committee of our hospital. As a retrospective study, the requirement for written informed consent was waived.

The inclusion criteria were as follows: (1) confirmed diagnosis of locally advanced unresectable hilar cholangiocarcinoma, as determined by a multidisciplinary team including radiologists and hepatobiliary surgeons according to NCCN guidelines (12); (2) expected survival of more than 3 months; (3) no prior history of external beam radiotherapy. Reasons for unresectability included medical inoperability, technically unresectable disease per institutional surgical criteria, ineligibility for transplantation, and patient refusal. The exclusion criteria were as follows: (1) allergy to iodinated contrast agents, precluding cholangiography; (2) complete biliary obstruction with impossible stent implantation on cholangiography; (3) severe dysfunction of vital organs, precluding treatment tolerance; (4) expected survival of less than 3 months; (5) poor compliance with treatment protocols.

Treatment allocation was determined based on institutional clinical practice protocols, technical availability, and patient preference. Before 2017, definitive external beam radiotherapy alone was the standard radiotherapy regimen for eligible patients in our center. After percutaneous ^192^Ir high-dose-rate brachytherapy was formally introduced in our department in early 2017, patients with suitable biliary anatomy, adequate liver function reserve, and willingness to receive the combined regimen were treated with EBRT plus brachytherapy boost. All treatment decisions were made after multidisciplinary evaluation and with full informed consent of patients.

### 2.2 Treatment method

**Figure 2:**
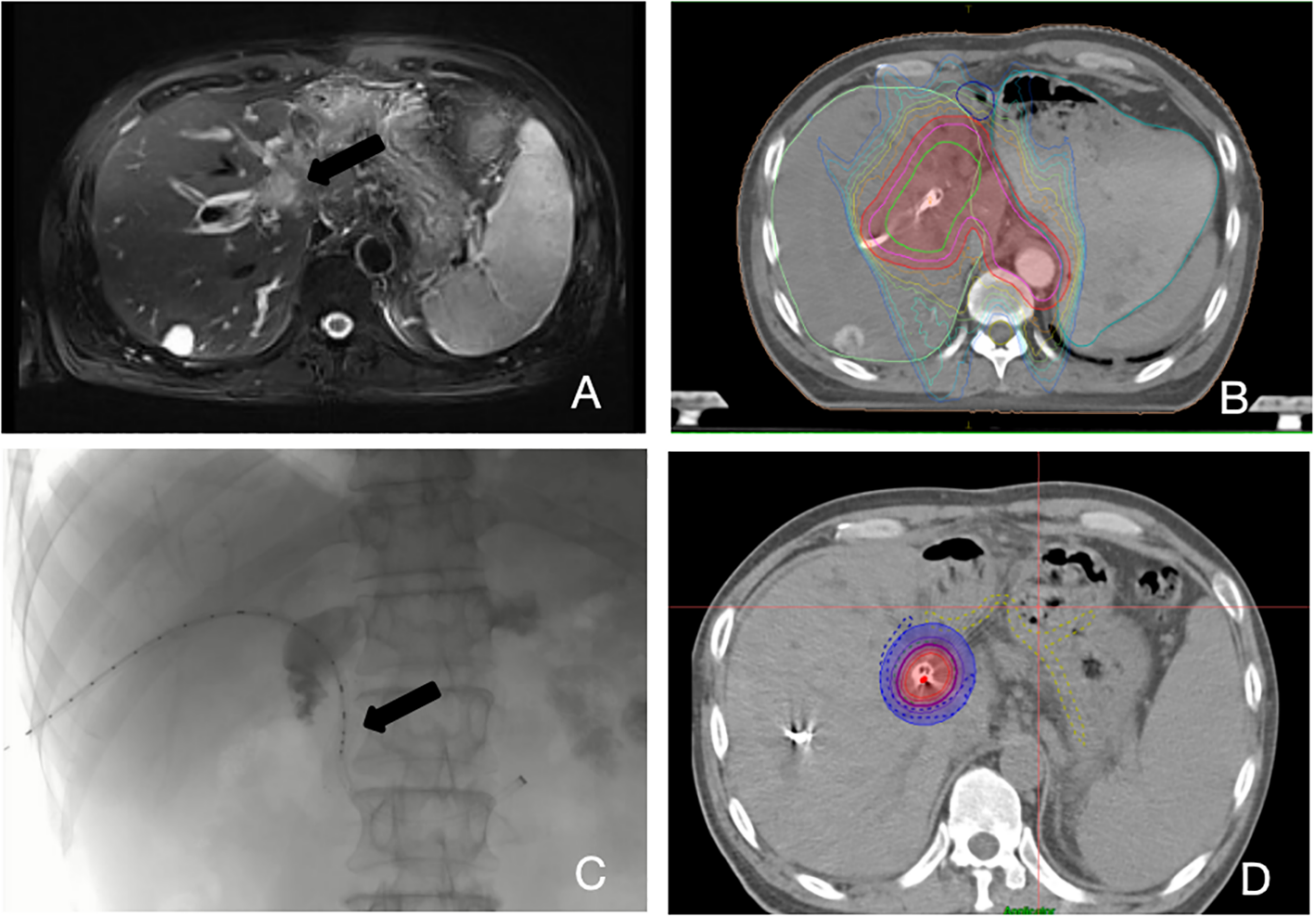
Representative images of the treatment procedure. **(A)** Contrast-enhanced CT image of type IV hilar cholangiocarcinoma, with the arrow indicating the tumor lesion; **(B)** fluoroscopic image after percutaneous biliary stent implantation; **(C)** fluoroscopic image of preplaced afterloading catheter with dummy source positioning, with the arrow indicating the biliary stent; **(D)** axial dose distribution map of ^192^Ir brachytherapy.

All patients first underwent percutaneous biliary stent implantation, followed by external beam radiotherapy. Patients in the ^192^Ir group additionally received ^192^Ir brachytherapy after completion of external beam radiotherapy (**Figure 2**). Concurrent chemotherapy was administered for 1 to 2 cycles during radiotherapy. After radiotherapy, most patients received 2 to 4 cycles of adjuvant chemotherapy, while a small proportion of patients did not receive adjuvant chemotherapy due to poor tolerance or early disease progression, with regimen selection based on performance status and liver function. Details of chemotherapy regimens are summarized in **Table 1**. For concurrent chemoradiotherapy, gemcitabine was administered at 1000 mg/m² on day 1 and day 8 every 3 weeks; capecitabine was administered at 825 mg/m² twice daily on radiotherapy days; the gemcitabine plus cisplatin (GC) regimen consisted of gemcitabine 1000 mg/m² on day 1 and cisplatin 25 mg/m² on day 1 to 3 every 3 weeks. Adjuvant chemotherapy followed the same dose principle, with dose adjustment based on adverse reactions.

**Table 1:** Distribution of chemotherapy regimens in the two groups.

Chemotherapy regimen	Ir-192 group (n=32) n (%)	EBRT group (n=30) n (%)	P value
Concurrent chemoradiotherapy phase			0.740
Gemcitabine + cisplatin (GC regimen)	7 (21.9)	5 (16.7)	
Single-agent gemcitabine	16 (50.0)	14 (46.7)	
Single-agent capecitabine	9 (28.1)	11 (36.7)	
Adjuvant chemotherapy phase			0.883
Gemcitabine + cisplatin (GC regimen)	15 (46.9)	13 (43.3)	
Single-agent gemcitabine	7 (21.9)	8 (26.7)	
Single-agent capecitabine	4 (12.5)	5 (16.7)	
No adjuvant chemotherapy	6 (18.8)	4 (13.3)	

Ir-192, iridium-192; EBRT, external beam radiation therapy; GC, gemcitabine plus cisplatin.

### 2.3 Percutaneous biliary stent implantation

The procedure was performed with the patient in the supine position, with oxygen inhalation, electrocardiogram monitoring, and preoperative analgesia. After routine disinfection and draping, the puncture site was selected at the 10th–11th intercostal space in the right midaxillary line. Under fluoroscopic guidance, a dilated peripheral bile duct was punctured after local anesthesia. After the puncture needle entered the dilated bile duct, the stylet was removed and contrast medium was injected for opacification of the biliary tree. A sheath was advanced over a microguidewire, retained bile was aspirated, and the bile duct was repeatedly irrigated with gentamicin saline solution. Repeat cholangiography was performed to delineate the common bile duct obstruction. After balloon dilation of the diseased bile duct, the lesion length and normal bile duct diameter were measured, an appropriate stent was deployed, and an internal-external drainage tube was placed.

### 2.4 External beam radiotherapy

All patients received intensity-modulated radiotherapy delivered by an Elekta Synergy medical linear accelerator. The prescribed dose to the planning target volume (PTV) was 45 Gy in 25 fractions of 1.8 Gy, 5 fractions per week, for the ^192^Ir group; and 60 Gy in 30 fractions of 2.0 Gy, 5 fractions per week, for the EBRT group. For simulation, patients were immobilized in the supine position with a low-temperature thermoplastic body shell on a dedicated radiotherapy positioning frame. Contrast-enhanced CT scans were acquired during quiet breathing, with a slice thickness of 5 mm. The scan range covered from the diaphragm to the lower pole of the kidney. Images were transferred to the Monaco 5.0 treatment planning system for target delineation and plan design.

The gross tumor volume (GTV) was defined on CT images, supplemented by abdominal MRI findings. The clinical target volume (CTV) was generated by expanding the GTV 0.5 cm radially and 1.0 cm craniocaudally, including peribiliary lymphatic drainage areas, pancreaticoduodenal and celiac lymph node basins, with appropriate adjustments to avoid overlapping with critical organs. The PTV was generated by expanding the CTV 1.0 cm in all directions. Organs at risk including the liver, small intestine, stomach, and spinal cord were also delineated. Plan requirements were as follows: 95% isodose line covering 100% of the PTV; liver mean dose < 20 Gy; duodenum V50 < 5%; small intestine maximum dose < 52 Gy and V45 < 195 cm³; stomach maximum dose < 54 Gy and V50 < 10%. All treatment plans met the above dose-volume constraints, and core dosimetric parameters of targets and organs at risk are presented in the results.

### 2.5 ^192^Ir brachytherapy

Brachytherapy was initiated after completion of external beam radiotherapy. First, an interventional fluoroscopic procedure was performed to preplace afterloading catheters. The patient was positioned supine, and a single 8 Fr vascular sheath was advanced into the biliary tract via percutaneous puncture under anesthesia. The tip of the catheter was positioned at the proximal side of the duodenal papilla. After insertion of the catheter and dummy source, the catheter position was adjusted and confirmed under fluoroscopy. The catheter and accessories were secured with sterile dressings to maintain stable position. For patients with type IV hilar cholangiocarcinoma, bilateral catheters were placed using the same technique.

Simulation CT scans were acquired and transferred to the afterloading radiotherapy planning system. The GTV was delineated, and the prescribed dose was 20 Gy in 4 fractions of 5 Gy. Treatment plans were generated with assessment of D100, D90, V100, and V90 for the GTV during plan optimization, and dose constraints for the duodenum, liver, stomach, and small intestine. The primary dosimetric endpoint for reporting was GTV D90. The safe dose range was defined by the D2cc of organs at risk, combined with the cumulative dose from external beam radiotherapy. After plan approval, treatment was delivered using a ^192^Ir high-dose-rate afterloader (MicroSelectron V3), twice weekly with a minimum interval of 48 hours between fractions. After brachytherapy, the radiotherapy catheters were removed and the biliary internal-external drainage tube was reinserted. Anti-inflammatory agents and nutritional support were provided after brachytherapy to prevent complications.

### 2.6 Efficacy evaluation and follow-up

Efficacy was evaluated according to the Response Evaluation Criteria in Solid Tumors version 1.1 (RECIST 1.1) (13). Contrast-enhanced CT or MRI of the upper abdomen was performed regularly after treatment. Imaging review was performed monthly during the first 3 months after treatment completion, and every 3 months thereafter. Survival status was observed and recorded. Progression-free survival (PFS) was defined as the time from initiation of radiotherapy to disease progression or death from any cause. Overall survival (OS) was defined as the time from initiation of radiotherapy to death from any cause. Short-term tumor response, PFS, and OS were calculated as study endpoints. Changes in total bilirubin (TBIL), direct bilirubin (DBIL), alanine aminotransferase (ALT), aspartate aminotransferase (AST), and carbohydrate antigen 19-9 (CA19-9) from baseline to 3 months after treatment were compared between the two groups. Radiation-related non-hematological adverse reactions were graded according to the Radiation Therapy Oncology Group (RTOG) acute radiation morbidity scoring criteria (14). Hematological toxicities were graded according to the National Cancer Institute Common Terminology Criteria for Adverse Events (NCI-CTCAE) version 5.0. Follow-up was conducted until July 2021. Stent patency was defined as the time from stent implantation to stent dysfunction or the last follow-up.

### 2.7 Statistical analysis

Statistical analysis was performed using SPSS version 26.0 software. Normally distributed quantitative data are expressed as mean ± standard deviation. Continuous variables were compared using independent samples t-test between groups and paired samples t-test for within-group comparisons. Non-normally distributed variables were compared using the Mann–Whitney U test. Categorical variables are expressed as numbers and percentages, and compared using Fisher’s exact test. Survival curves were estimated using the Kaplan–Meier method and compared using the log-rank test. Multivariate Cox proportional hazards regression analysis was performed to identify independent prognostic factors for PFS and to adjust for potential confounding factors between treatment groups, including age, sex, Bismuth-Corlette type, TNM stage, Child-Pugh class, chemotherapy regimen, and baseline CA19–9 level, to mitigate the impact of selection bias. Subgroup analysis stratified by chemotherapy regimen was not conducted in this study, as stratification of the limited sample size would further reduce the number of events in each subgroup, resulting in insufficient statistical power and unreliable effect estimates. A two-sided P value < 0.05 was considered statistically significant.

## 3 Results

### 3.1 Baseline characteristics

From January 2014 to March 2019, 62 patients with pathologically and/or clinically confirmed unresectable hilar cholangiocarcinoma treated with radiotherapy combined with biliary stenting at our hospital were included. Patients were divided into the ^192^Ir group (n=32) and the EBRT group (n=30). Demographic and disease characteristics were balanced between the two groups, with no statistically significant differences in baseline parameters (**Table 2**).

**Table 2:** Baseline clinical characteristics of the two groups.

Characteristics	Ir-192 group (n=32)	EBRT group (n=30)	P value
Demographics			
Age, years, mean ± SD	62.3 ± 8.5	61.5 ± 9.1	0.725
Male sex, n (%)	24 (75.0)	23 (76.7)	0.878
ECOG performance status, n (%)			0.856
0–1	25 (78.1)	24 (80.0)	
2	7 (21.9)	6 (20.0)	
Tumor features			
Bismuth-Corlette classification, n (%)			0.882
Type II	3 (9.4)	4 (13.3)	
Type III	22 (68.7)	20 (66.7)	
Type IV	7 (21.9)	6 (20.0)	
AJCC TNM stage (8th edition), n (%)			0.900
IIIA	14 (43.7)	12 (40.0)	
IIIB	11 (34.4)	12 (40.0)	
IIIC	7 (21.9)	6 (20.0)	
Portal vein invasion, n (%)	19 (59.4)	17 (56.7)	0.829
Child-Pugh class, n (%)			0.830
A	26 (81.2)	25 (83.3)	
B	6 (18.8)	5 (16.7)	
Baseline laboratory parameters			
TBIL, μmol/L, mean ± SD	251.47 ± 85.79	232.69 ± 86.89	0.395
CA19-9, U/mL, mean ± SD	443.36 ± 213.44	461.17 ± 206.98	0.740

Ir-192, iridium-192; EBRT, external beam radiation therapy; ECOG, Eastern Cooperative Oncology Group; AJCC, American Joint Committee on Cancer; TBIL, total bilirubin; CA19-9, carbohydrate antigen 19-9.

### 3.2 Radiotherapy dosimetry

Details of radiotherapy dosimetric parameters and biologically effective dose (EQD2) are summarized in **Table 3**. The total EQD2 to the GTV (α/β = 10 Gy) was higher in the ^192^Ir group than in the EBRT group, while the cumulative EQD2 to organs at risk (α/β = 3 Gy) was comparable between the two groups.

**Table 3:** Comparison of radiotherapy dosimetric parameters and biologically effective dose.

Parameter	Ir-192 group (n=32)	EBRT group (n=30)
External beam radiotherapy		
Prescribed dose, Gy/fx	45/1.8	60/2.0
PTV D90, Gy	45.8 ± 1.2	61.1 ± 1.5
^192^Ir brachytherapy		
Prescribed dose, Gy/fx	20/5.0	—
GTV D90, Gy	21.3 ± 2.1	—
Total EQD2 (α/β = 10 Gy), tumor		
GTV total EQD2, Gy	71.6 ± 3.5	61.2 ± 1.6
Total EQD2 (α/β = 3 Gy), organs at risk		
Liver Dmean, Gy	18.2 ± 2.4	19.5 ± 2.1
Duodenum D2cc, Gy	52.7 ± 3.6	54.2 ± 2.9
Spinal cord Dmax, Gy	38.1 ± 2.2	41.3 ± 1.8

EQD2, biologically effective dose normalized to 2 Gy per fraction; GTV, gross tumor volume; PTV, planning target volume; D90, dose received by 90% of the target volume; Dmean, mean dose; D2cc, dose received by 2 cc of the organ; Dmax, maximum dose.

### 3.3 Efficacy and survival outcomes

Follow-up was completed in July 2021. The follow-up duration ranged from 7 to 56 months, with a median follow-up of 16.3 months. At the end of follow-up, 5 patients remained alive (3 in the ^192^Ir group, 2 in the EBRT group) and 57 patients had died. The primary cause of death was tumor progression, including liver metastasis in 28 patients, lung metastasis in 18 patients, and combined liver and lung metastasis in 11 patients. Distant metastasis was the predominant failure pattern in both groups. As local-only progression was not predefined as an independent study endpoint and was not uniformly documented during retrospective follow-up, formal local control rate was not calculated in this study. No treatment-related death was observed in either group. Short-term efficacy was evaluated at 3 months after treatment completion (**Table 4**). In the ^192^Ir group, 5 patients achieved complete response (CR, 15.6%), 25 achieved partial response (PR, 78.1%), 2 had stable disease (SD, 6.3%), and no patient had progressive disease (PD), with an objective response rate (ORR) of 93.8%. In the EBRT group, 2 patients achieved CR (6.7%), 19 achieved PR (63.3%), 8 had SD (26.7%), and 1 had PD (3.3%), with an ORR of 70.0%. The ORR was significantly higher in the ^192^Ir group than in the EBRT group (P = 0.020). The DCR was 100.0% in the ^192^Ir group and 96.7% in the EBRT group, with no statistically significant difference between groups (P = 0.484).

**Table 4:** Short-term efficacy evaluation at 3 months after treatment (RECIST 1.1).

Efficacy endpoint	Ir-192 group (n=32) n (%)	EBRT group (n=30) n (%)	P value
Complete response (CR)	5 (15.6)	2 (6.7)	
Partial response (PR)	25 (78.1)	19 (63.3)	
Stable disease (SD)	2 (6.3)	8 (26.7)	
Progressive disease (PD)	0 (0.0)	1 (3.3)	
Objective response rate (ORR, CR+PR)	30 (93.8)	21 (70.0)	0.020
Disease control rate (DCR, CR+PR+SD)	32 (100.0)	29 (96.7)	0.484

CR, complete response; PR, partial response; SD, stable disease; PD, progressive disease; ORR, objective response rate; DCR, disease control rate.

**Figure 3:**
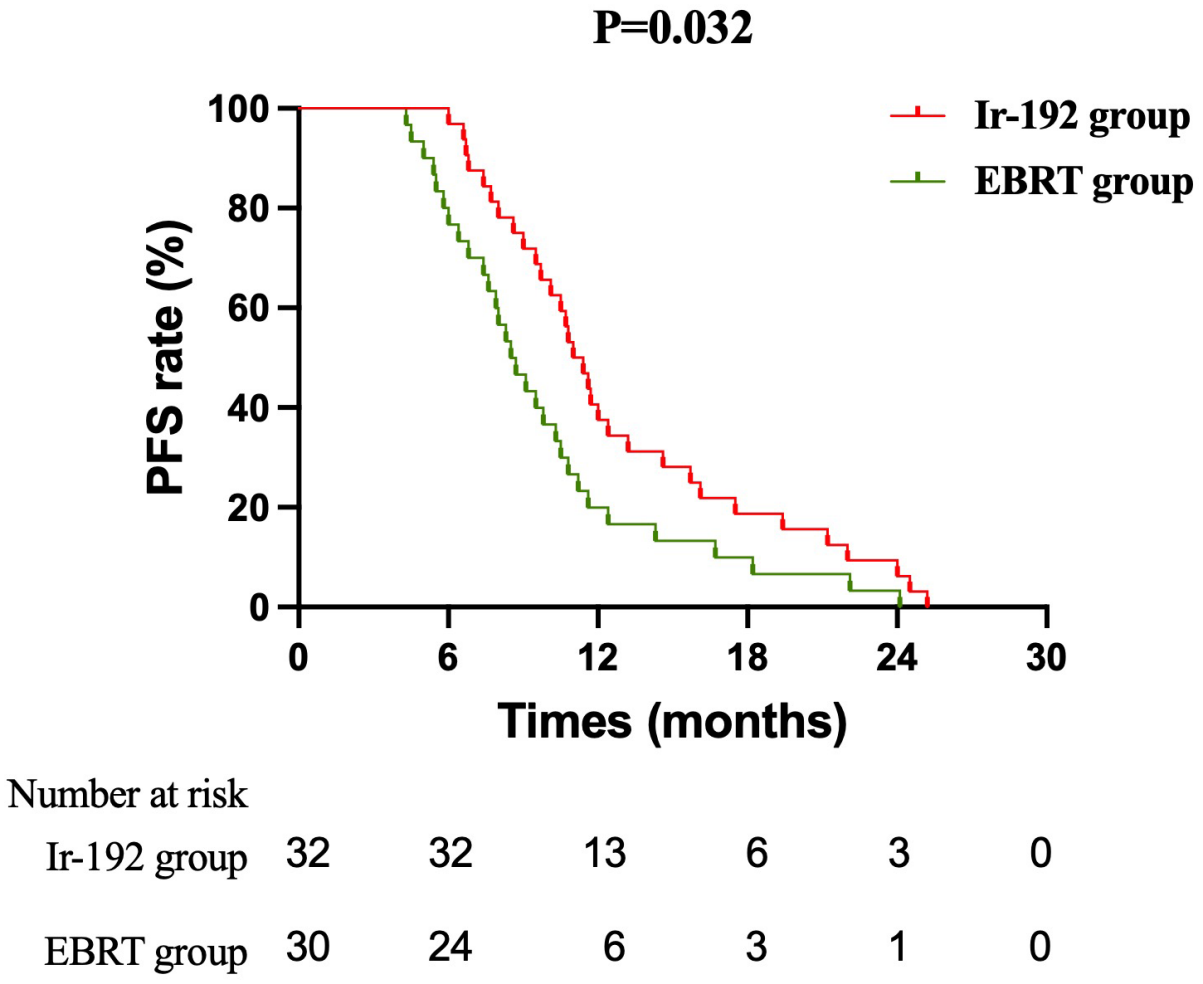
Kaplan–Meier curves of progression-free survival for the two groups.

**Figure 4:**
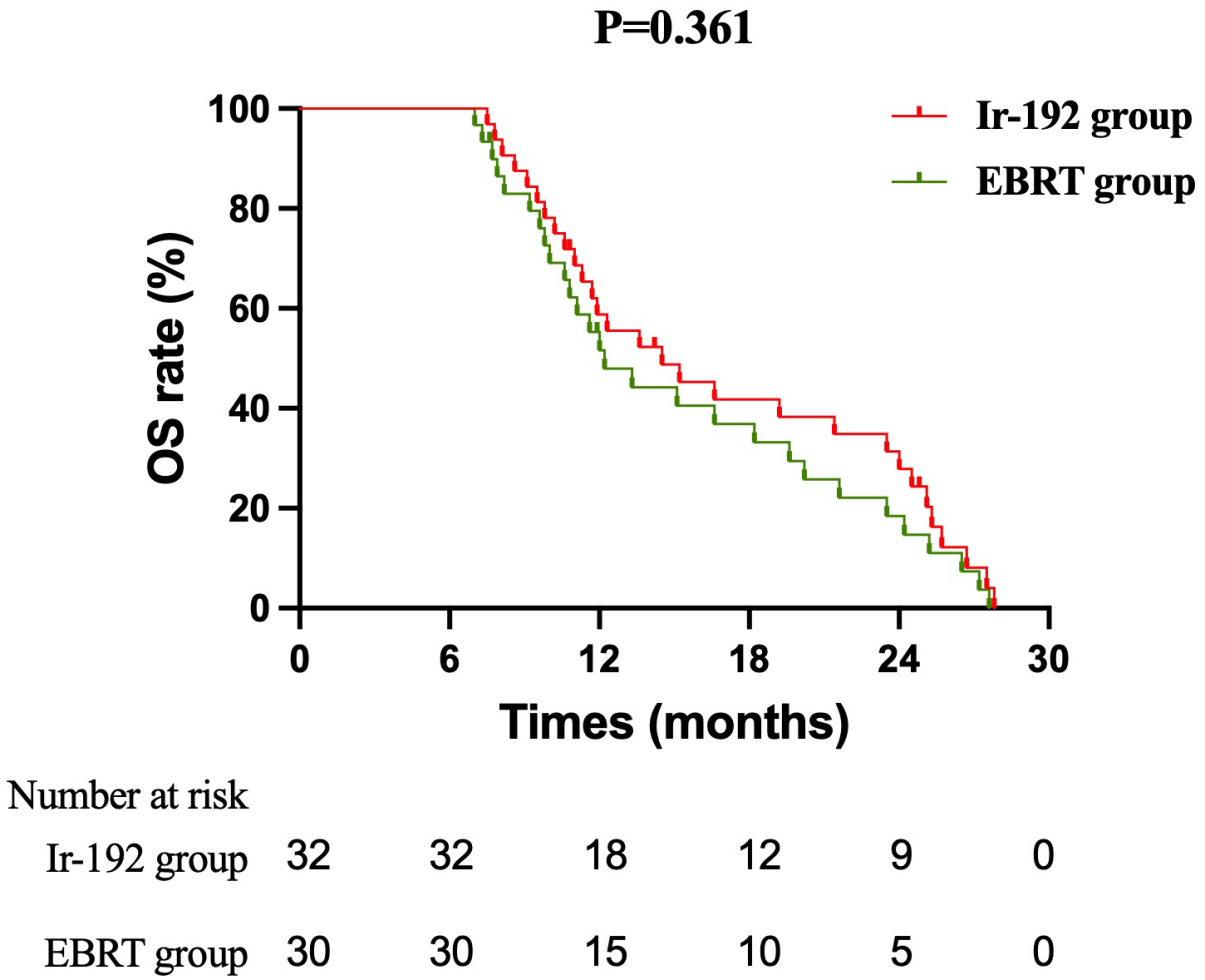
Kaplan–Meier curves of overall survival for the two groups.

The median PFS was 11.2 months in the ^192^Ir group and 8.6 months in the EBRT group. The 1-year PFS rates were 40.6% and 20.0%, and the 2-year PFS rates were 9.4% and 3.3%, respectively. The difference in PFS between the two groups was statistically significant (P = 0.032; **Figure 3**). The median OS was 14.5 months in the ^192^Ir group and 12.2 months in the EBRT group. The 1-year OS rates were 56.3% and 50.0%, and the 2-year OS rates were 28.1% and 16.7%, respectively. No statistically significant difference in OS was observed between the two groups (P = 0.361; **Figure 4**). After adjustment for chemotherapy regimen and other baseline prognostic factors, Multivariate Cox regression analysis showed that treatment with ^192^Ir brachytherapy was an independent protective factor for PFS (hazard ratio = 0.56, 95% confidence interval 0.32–0.97, P = 0.039). Bismuth-Corlette type IV and higher TNM stage were independent risk factors for PFS (**Table 5**). With regard to stent patency, the median stent patency time was 9.8 months in the ^192^Ir group and 7.3 months in the EBRT group (P = 0.027). The 6-month and 12-month stent patency rates were 78.1% and 37.5% in the ^192^Ir group, and 56.7% and 20.0% in the EBRT group, respectively.

**Table 5:** Multivariate Cox regression analysis of progression-free survival.

Variable	HR	95% CI	P value
Treatment (Ir-192 + EBRT vs EBRT alone)	0.56	0.32 – 0.97	0.039
Age (> 65 vs ≤ 65 years)	1.12	0.64 – 1.96	0.692
Male sex	0.94	0.53 – 1.67	0.837
Bismuth-Corlette type (IV vs II–III)	1.85	1.01 – 3.39	0.046
TNM stage (IIIC vs IIIA–IIIB)	2.13	1.15 – 3.94	0.016
Child-Pugh class (B vs A)	1.58	0.82 – 3.04	0.171
Chemotherapy (GC vs single agent)	0.83	0.47 – 1.47	0.524
Baseline CA19-9 (per 100 U/mL increase)	1.08	0.99 – 1.18	0.072

HR, hazard ratio; CI, confidence interval; GC, gemcitabine plus cisplatin.

### 3.4 Laboratory parameters

Levels of TBIL, DBIL, ALT, AST, and CA19–9 decreased significantly from baseline to 3 months after treatment in both groups (all P < 0.001). At 3 months after treatment, levels of all five parameters were significantly lower in the ^192^Ir group than in the EBRT group, with statistically significant differences (**Table 6**).

**Table 6:** Comparison of liver function and CA19–9 levels before and 3 months after treatment between the two groups.

Indicator	Time point	Ir-192 group (n=32)	EBRT group (n=30)	P value
TBIL, μmol/L	Before treatment	251.47 ± 85.79	232.69 ± 86.89	0.395
	3 months after	32.94 ± 19.19	53.49 ± 25.77	0.001
DBIL, μmol/L	Before treatment	204.38 ± 67.81	199.46 ± 64.42	0.771
	3 months after	27.95 ± 16.54	39.73 ± 25.07	0.032
ALT, U/L	Before treatment	161.31 ± 54.69	176.40 ± 52.45	0.273
	3 months after	31.47 ± 13.13	48.13 ± 16.02	< 0.001
AST, U/L	Before treatment	174.03 ± 59.28	190.97 ± 55.22	0.250
	3 months after	32.81 ± 17.82	44.17 ± 17.59	0.014
CA19-9, U/mL	Before treatment	443.36 ± 213.44	461.17 ± 206.98	0.740
	3 months after	203.72 ± 117.21	268.03 ± 124.15	0.040

TBIL, total bilirubin; DBIL, direct bilirubin; ALT, alanine aminotransferase; AST, aspartate aminotransferase; CA19-9, carbohydrate antigen 19-9.

### 3.5 Adverse reactions

No statistically significant difference was found in the incidence of adverse reactions between the two groups (**Table 7**). Most adverse events were grade 1–2, and resolved after symptomatic management. No severe complications such as biliary fistula or biliary bleeding occurred in either group during treatment. In the ^192^Ir group, grade 1–2 neutropenia occurred in 15 patients (46.8%), and grade ≥ 3 neutropenia in 4 patients (12.5%). In the EBRT group, grade 1–2 neutropenia occurred in 13 patients (43.3%), and grade ≥ 3 neutropenia in 4 patients (13.3%). Grade 1–2 thrombocytopenia occurred in 13 patients (40.6%) in the ^192^Ir group and 12 patients (40.0%) in the EBRT group, with grade ≥ 3 events in 5 (15.6%) and 3 (10.0%) patients, respectively. Grade 1–2 nausea and vomiting occurred in 11 patients (34.4%) in the ^192^Ir group and 10 patients (33.3%) in the EBRT group. Grade 1–2 abdominal pain was reported in 9 patients (28.1%) in the ^192^Ir group and 9 patients (30.0%) in the EBRT group. Cholangitis occurred in 3 patients (9.4%) in the ^192^Ir group and 1 patient (3.3%) in the EBRT group, and resolved after anti-infective treatment.

**Table 7:** Comparison of adverse reactions between the two groups.

Adverse reaction	Grade	Ir-192 group (n=32) n (%)	EBRT group (n=30) n (%)	P value
Hematological toxicities				
Neutropenia	1–2	15 (46.8)	13 (43.3)	0.962
	≥ 3	4 (12.5)	4 (13.3)	
Thrombocytopenia	1–2	13 (40.6)	12 (40.0)	0.773
	≥ 3	5 (15.6)	3 (10.0)	
Non-hematological toxicities				
Nausea and vomiting	1–2	11 (34.4)	10 (33.3)	0.931
	≥ 3	0 (0.0)	0 (0.0)	
Abdominal pain	1–2	9 (28.1)	9 (30.0)	0.871
	≥ 3	0 (0.0)	0 (0.0)	
Cholangitis	1–2	3 (9.4)	1 (3.3)	0.652
	≥ 3	0 (0.0)	0 (0.0)	
Biliary bleeding	Any	0 (0.0)	0 (0.0)	—
Biliary fistula	Any	0 (0.0)	0 (0.0)	—

Data are presented as n (%). Non-hematological adverse reactions were graded according to the RTOG acute radiation morbidity scoring criteria; hematological toxicities were graded according to CTCAE 5.0 criteria. Comparisons between groups were performed using Fisher’s exact test.

RTOG, Radiation Therapy Oncology Group; Ir-192, iridium-192; EBRT, external beam radiation therapy.

## 4 Discussion

^192^Ir high-dose-rate brachytherapy differs from the more widely reported ^125^I seed implantation brachytherapy. It delivers a steep dose gradient around the source, allowing high dose delivery to the tumor target with limited exposure to surrounding organs at risk, and no residual radioactive material remains in the body after treatment. It is widely used as a boost modality after external beam radiotherapy to enhance local tumor control. A 2021 systematic review including 23 studies confirmed that intraluminal HDR ^192^Ir brachytherapy combined with biliary stenting was associated with prolonged stent patency (median 10 months vs 4–6 months with stenting alone), improved tumor response rates, and a trend toward better local tumor control, with overall acceptable toxicity (15). A pooled analysis of three Italian centers further demonstrated that EBRT combined with intraluminal ^192^Ir brachytherapy boost yielded favorable survival in unresectable extrahepatic cholangiocarcinoma, with a median overall survival of 12.6 months across heterogeneous patient cohorts (16). In clinical practice, the ^192^Ir brachytherapy boost strategy has been most thoroughly validated in neoadjuvant settings before liver transplantation, as represented by the Mayo Clinic protocol. This standard regimen consists of 45 Gy EBRT with concurrent fluoropyrimidine chemotherapy, followed by 16–30 Gy intraluminal brachytherapy boost, and has achieved 5-year survival rates exceeding 70% in carefully selected unresectable patients (17, 18). For patients ineligible for transplantation, dose escalation via brachytherapy boost remains a key strategy to improve tumor control on the basis of definitive EBRT. A single-center study from India adopted a similar regimen of 45 Gy EBRT plus 14–20 Gy HDR brachytherapy boost for unresectable hilar cholangiocarcinoma, reporting a median overall survival of 13.2 months, which was superior to historical data of definitive EBRT alone at the same institution (19). A phase I dose-escalation study also identified a dose-dependent trend in tumor response and stent patency for intraluminal HDR brachytherapy, supporting the rationale of dose escalation via this modality (20). Previous studies have mostly reported the use of ^125^I seed strands for unresectable hilar cholangiocarcinoma (21), while head-to-head comparative data between percutaneous ^192^Ir brachytherapy boost plus EBRT and standard-dose definitive EBRT alone remain relatively scarce in non-transplant candidates.

Kamphues et al. (22) retrospectively analyzed 10 patients with intrahepatic or hilar cholangiocarcinoma recurrence after resection, all treated with at least one session of CT-guided ^192^Ir brachytherapy. The 1-year survival rate after treatment was 77.1%, with no severe complications reported, suggesting a favorable safety profile. Mukewar et al. (23) reported that external beam radiotherapy combined with endoscopic ^192^Ir brachytherapy could downstage unresectable perihilar cholangiocarcinoma to meet liver transplantation criteria as a neoadjuvant regimen. However, endoscopic retrograde catheter placement is technically challenging and associated with adverse events such as cholangitis, duodenal injury, and severe abdominal pain. A study from the Mayo Clinic enrolled 25 patients with unresectable hilar cholangiocarcinoma treated with external beam radiotherapy plus ^192^Ir brachytherapy and 5-fluorouracil chemotherapy, followed by liver transplantation in 11 patients. Among 8 patients with follow-up longer than 12 months, the median follow-up was 44 months (17). However, liver transplantation has limited accessibility and high cost in clinical practice. Ba et al. (24) showed that compared with endoscopic retrograde cholangiopancreatography, percutaneous transhepatic biliary drainage was associated with lower incidence of cholangitis and pancreatitis in patients with type II, III, and IV hilar cholangiocarcinoma, especially a reduced risk of cholangitis in type III and IV disease, and lower hospitalization costs, making it the preferred interventional drainage approach. Several studies (25, 26) have shown that percutaneous biliary stenting combined with brachytherapy or external beam radiotherapy is a safe and effective palliative treatment for unresectable hilar cholangiocarcinoma, as biliary stenting can effectively relieve jaundice.

External beam radiotherapy alone for hilar cholangiocarcinoma is often limited by dose constraints to adjacent organs such as the liver and duodenum. In the present study, we compared two routinely used clinical radiotherapy strategies: moderate-dose EBRT plus ^192^Ir HDR brachytherapy boost, and standard-dose definitive EBRT alone, rather than evaluating the isolated effect of brachytherapy on the basis of identical EBRT background. Dosimetric analysis showed that the combined regimen delivered a higher biologically effective dose to the tumor target while maintaining comparable dose exposure to organs at risk, compared with external beam radiotherapy alone. Given this study design, the observed progression-free survival benefit cannot be unambiguously attributed to the brachytherapy technique alone; the higher cumulative tumor biological dose, the steep dose gradient characteristic of brachytherapy, or a combination of both factors may contribute to the improved outcomes. This dosimetric profile is consistent with the core rationale of brachytherapy boost, which aims to escalate tumor dose without exceeding the tolerance of surrounding normal tissues. Consistent with our findings, multiple retrospective cohort studies have demonstrated that tumor dose escalation via brachytherapy boost correlates with improved short-term tumor response and prolonged progression-free survival, without significantly increasing normal tissue toxicity (16, 19). A recent retrospective study of definitive chemoradiotherapy for biliary tract cancer reported consistent findings that tumor dose escalation was associated with improved local control and PFS in unresectable perihilar cholangiocarcinoma (27). Given that local control was not evaluated as a separate endpoint in this study, we cannot conclude that the PFS benefit stems exclusively from improved local tumor control. In addition, more favorable short-term tumor response may contribute to longer stent patency and better relief of biliary obstruction, which corresponds to the greater reduction in bilirubin and liver enzyme levels observed in the ^192^Ir group at 3 months. The survival outcomes observed in our cohort are comparable to those reported in previous non-transplant brachytherapy series, and the 14.5-month median overall survival in the combined treatment group falls within the upper range of reported data for unresectable hilar cholangiocarcinoma treated with chemoradiotherapy. This study further supplements real-world evidence for percutaneous transhepatic HDR brachytherapy boost in a non-transplant population, which remains less extensively reported than endoscopic approaches and neoadjuvant transplant protocols.

A population-based study using the SEER database reported that the 1-year and 2-year OS rates of patients with hilar cholangiocarcinoma were 26.2% and 10.7%, respectively, lower than those of intrahepatic cholangiocarcinoma and hepatocellular carcinoma, indicating a poor prognosis (28). Patients with Bismuth-Corlette type III and IV disease are often inoperable and have even worse outcomes. Current palliative treatment options include biliary drainage, chemotherapy, radiotherapy, photodynamic therapy, and supportive care. Pietge et al. (29) reported a dose-finding study of systemic gemcitabine plus cisplatin combined with hepatic arterial infusion of floxuridine in 12 patients with unresectable cholangiocarcinoma, showing partial response in 27% of patients and stable disease in 73% at 3 months. In comparison, the PR rate in the present study was higher, which may be partly explained by the addition of local radiotherapy and the relatively small sample size of the aforementioned study. Cassani et al. (30) reported that adequate biliary decompression was associated with prolonged OS, with a median OS of 11.0 months and 1-year OS rate of 48%, and successful stent placement was significantly correlated with longer survival. Shin et al. (31) reported that gemcitabine plus cisplatin chemotherapy yielded a median OS of 12.8 months in advanced hilar cholangiocarcinoma, compared with 6.1 months in the best supportive care group. Bisello et al. (32) reported a retrospective study of chemoradiotherapy for unresectable biliary cancer, with a median OS of 13.5 months, and 1-year and 2-year OS rates of 58.1% and 25.8%, respectively. The survival outcomes of the ^192^Ir group in the present study are comparable to or slightly higher than these previously published data.

In the present study, the difference in OS between the two groups did not reach statistical significance. Several factors may contribute to this finding. First, the sample size of this single-center retrospective study was relatively small, resulting in limited statistical power to detect a difference in OS. Second, distant metastasis was the main cause of death in this cohort, and improved short-term tumor response may not readily translate into overall survival benefit in a disease with high metastatic potential. Third, subsequent treatment after progression was not controlled for between groups, which may have confounded OS results. All adverse reactions were manageable with symptomatic treatment, and no severe procedure-related complications such as biliary fistula or biliary bleeding occurred, indicating a tolerable safety profile of the combined regimen.

This study has several inherent limitations associated with its retrospective, non-randomized design, which are systematically acknowledged below. First, this was a single-center retrospective study with non-randomized treatment allocation, and potential selection bias cannot be fully excluded. Although multivariable adjustment was performed to control for measurable confounding factors, unmeasured confounders may still affect the results. Given the limited overall sample size, propensity score matching or inverse probability weighting was not adopted in this study, as further reduction of effective sample size would substantially decrease statistical power and lead to unstable estimates. Second, chemotherapy regimens were heterogeneous in both concurrent and adjuvant settings, including single-agent gemcitabine, single-agent capecitabine, and gemcitabine plus cisplatin combination therapy, and a proportion of patients did not receive adjuvant chemotherapy due to poor tolerance or early disease progression. This heterogeneity reflects individualized treatment decision-making in real-world clinical practice, where regimen selection is based on patient performance status, liver function reserve, and treatment tolerance, but it may introduce confounding effects on survival outcomes independent of radiotherapy strategies. To address this issue, we included chemotherapy regimen as a covariate in the multivariate Cox model to adjust for its potential impact. Nevertheless, residual confounding cannot be completely excluded, and the modifying effect of different chemotherapy regimens on the efficacy of different radiotherapy strategies cannot be fully clarified in this study. Third, the two groups received distinct radiotherapy strategies rather than uniform EBRT with or without brachytherapy, and the cumulative biologically effective dose to the tumor was higher in the combined treatment group. Therefore, the independent contribution of the brachytherapy technique itself and the higher tumor dose to the observed survival benefit cannot be fully disentangled; the findings of this study reflect the overall effect of this combined radiotherapy strategy. Dosimetric analysis confirmed that the combined regimen achieved higher tumor dose without increasing normal tissue exposure. Fourth, data on stent patency and quality of life were not systematically collected. Future studies with larger sample sizes and prospective randomized designs are warranted to validate these findings.

## 5 Conclusion

This retrospective analysis shows that the strategy of moderate-dose external beam radiotherapy combined with ^192^Ir brachytherapy boost, together with biliary stenting, is associated with improved short-term tumor response and prolonged progression-free survival in patients with unresectable hilar cholangiocarcinoma, with a trend toward prolonged overall survival that does not reach statistical significance. The regimen has a tolerable safety profile, and may serve as a feasible locoregional treatment option for this patient population. However, given the limitations of this study, particularly the single-center retrospective design and moderate sample size, the conclusions require validation in prospective randomized controlled trials. Future research should aim to optimize the dose and fractionation of brachytherapy, and to systematically evaluate its impact on long-term survival, stent patency, and quality of life.

## Data Availability

The original contributions presented in the study are included in the article/supplementary material. Further inquiries can be directed to the corresponding author.
